# Role of protein arginine methyltransferase 5 in inflammation and migration of fibroblast‐like synoviocytes in rheumatoid arthritis

**DOI:** 10.1111/jcmm.13020

**Published:** 2016-11-17

**Authors:** Dongying Chen, Shan Zeng, Mingcheng Huang, Hanshi Xu, Liuqin Liang, Xiuyan Yang

**Affiliations:** ^1^ Department of RheumatologyThe First Affiliated Hospital of Sun Yat‐sen UniversityGuangzhouChina

**Keywords:** rheumatoid arthritis, protein arginine methyltransferases 5, fibroblast‐like synoviocytes, migration, inflammation

## Abstract

To probe the role of protein arginine methyltransferase 5 (PRMT5) in regulating inflammation, cell proliferation, migration and invasion of fibroblast‐like synoviocytes (FLSs) from patients with rheumatoid arthritis (RA). FLSs were separated from synovial tissues (STs) from patients with RA and osteoarthritis (OA). An inhibitor of PRMT5 (EPZ015666) and short interference RNA (siRNA) against PRMT5 were used to inhibit PRMT5 expression. The standard of protein was measured by Western blot or immunofluorescence. The excretion and genetic expression of inflammatory factors were, respectively, estimated by enzyme‐linked immunosorbent assay (ELISA) and real‐time polymerase chain reaction (PCR). Migration and invasion *in vitro* were detected by Boyden chamber assay. FLSs proliferation was detected by BrdU incorporation. Increased PRMT5 was discovered in STs and FLSs from patients with RA. In RA FLSs, the level of PRMT5 was up‐regulated by stimulation with IL‐1β and TNF‐α. Inhibition of PRMT5 by EPZ015666 and siRNA‐mediated knockdown reduced IL‐6 and IL‐8 production, and proliferation of RA FLSs. In addition, inhibition of PRMT5 decreased *in vitro* migration and invasion of RA FLSs. Furthermore, EPZ015666 restrained the phosphorylation of IκB kinaseβ and IκBα, as well as nucleus transsituation of p65 as well as AKT in FLSs. PRMT5 regulated the production of inflammatory factors, cell proliferation, migration and invasion of RA FLS, which was mediated by the NF‐κB and AKT pathways. Our data suggested that targeting PRMT5 to prevent synovial inflammation and destruction might be a promising therapy for RA.

## Introduction

Rheumatoid arthritis (RA) is a chronic disease, which is characterized by the hyperplasia of synovial tissues (STs) and progressive destruction of articular cartilage, bone and ligaments 1. Inflammatory factors were essential in the initiation and development of synovial inflammation in RA 2. Moreover, fibroblast‐like synoviocytes (FLSs) in the synovial intimal lining played a central role in RA pathogenesis 3. Stable, activated RA FLSs exhibited tumour‐like characteristics 4, 5 and were critical in the development of pannus through migrating and invading to the cartilage and bone 6, 7, 8. Therefore, modulation of synovial inflammation and the aggressive behaviours of activated FLSs was a novel therapeutic strategy for RA.

Arginine methylation is an abundant post‐translational modification that occurs in mammalian cells and is catalysed by proteins of the arginine methyltransferase (PRMT) family. PRMT5, a member of PRMT family, is localized to both the cytoplasm and the nucleus of mammalian cells 9, 10. PRMT5 participates in many cell processes, including transcriptional repression 11, RNA splicing 12, signal transduction 13 and piRNA pathway activation 14. Overexpression of PRMT5 has been found in many kinds of tumours, including leukaemia 15, lymphoma 16, lung cancer 17, colorectal cancer 18 and breast cancer 19. The overexpression of PRMT5 was associated with tumour cell growth. Recent studies also indicated that PRMT5 was a key enzyme in regulating endothelial cell inflammation, cell proliferation as well as differentiation 20. However, it was still unclear whether PRMT5 was involved in RA. We hypothesized that PRMT5 might regulate the inflammation and invasive behaviours in RA FLSs. To investigate the validity of the hypothesis, we first examined the expression of PRMT5 in STs and FLSs. We then used a PRMT5 inhibitor and siRNA to study the influence of PRMT5 on the inflammation, cell proliferation, migration and invasion of RA FLSs. Finally, we verified the signal pathway involved in this process.

## Materials and methods

### Patients and isolation of FLSs

STs were gained from RA patients or osteoarthritis (OA) patients, who went through synovectomy or joint arthroplasty. Briefly, STs were incised into pieces by pieces, then digested with collagenase I (Sigma‐Aldrich, St. Louis, MO, USA) in DMEM/F12 medium (Invitrogen,Carlsbad, CA, USA) for 3 hrs at 37°C, 5% CO_2_. After centrifugation, the cells were fostered in DMEM/F12 medium that containing 10% FBS, 100 U/ml penicillin and 100 μg/ml strep at 37°C in a constant humidified incubator of 5% CO_2_, 21% O_2_ and 75% N_2_. The cells were trypsinized when the cells reached assemble and transited. The cells at passages of 3–7 were used in this study. Normal control FLSs were isolated from STs obtained by arthroscopic biopsy from patients that had menisci injuries without history of acute or chronic arthritis, which were kind gifts from Prof. Hanshi Xu in the First Affiliated Hospital of Sun Yat‐Sen University. The study was sanctified by the Medical Ethical Committee of the First Affiliated Hospital, SYSU, China, and executed on the basis of the Declaration of Helsinki.

### Immunohistochemical (IHC) analysis

STs were placed in 10% formalin overnight, paraffin‐imbed, incised into 5‐μm sections and put to 3‐aminopropyltriethoxysilane‐coated slides. Then, the sections were deparaffinized by xylene and ethanol which was rehydrated, and using microwave heating with antigen retrieval. Endogenous peroxidase activity was then inhibited by hatching the parts in 1% hydrogen peroxide about 30 min. The segment were then fostered with main antibodies against PRMT5 (Abcam, Cambridge, UK) at 4°C overnight. Then, the samples was washed three times with PBS for 5 min. each and hatched with the respective horseradish peroxidase (HRP)‐conjugated secondary antibodies, substrates and counterstained with haematoxylin.

### Transfection of siRNA

For the transfection of PRMT5 siRNA (Santa Cruz, California, USA), FLSs were fostered in six‐well plates; 100 nM siRNA and 10 mg/ml lipofectin in serum‐free medium as a transfection mixture was added to medium‐aspirated cells for 6 hrs. Then, the cells before experiments were incubated with total including 10% FBS DMEM/F12 for 48 hrs.

### *In vitro* migration and invasion assay of FLSs

Chemotaxis analysis of FLSs was preformed through the transwell (Corning, New York, NY, USA) migration assay. Filled with DMEM/F12 medium containing 10% FBS, the bottom chambers were used as a chemical enticement. The top chambers were cultured in 200 μl DMEM/F12 medium (without FBS) and FLSs with PRMT5‐specific inhibitor EPZ015666 (Selleck Chemicals, Houston, USA) or FLSs transfected with PRMT5 siRNA for 48 hrs. After 12 hrs, through a cotton swab, the cells which had not migrated were removed from the filter top. The migrated cells, on the bottom of the membrane, were immersed in methanol and dyed with 0.1% Crystal Violet. Through the ZEISS digital microscope dealing with images, we counted the stained cells for each analysis via the mean number of cells per five random areas. The *in vitro* invasion test was performed by inserting a Matrigel basement membrane matrix (BD Biosciences, Oxford, UK). Under a microscope, numbers of invaded cells were randomly selected and counted in 10 high‐power fields. These experiments were performed three times.

### Wounding migration

Wounding migration assays were performed as described previously 21. Briefly, RA FLSs were plated to confluence on 35‐mm culture dishes at a density of 2 × 10^5^ cells/ml, where 90% confluence allowed one parallel wound. The following day, wounds were created in a cell monolayer using 1‐ml sterile micropipette tips. Then starving media were used to wash detached cells, and RA FLSs were treated with or without 10% FBS. After 24 hrs of incubation, migration was quantified with Image J software by counting the cells that moved beyond a reference line.

### Proliferation assays

5‐Ethynyl‐2′‐deoxyuridine (EdU) is a thymidine analogue; when cells are dividing, it is incorporated into replicating DNA and then applied to sign proliferating cells. RA FLSs were trypsinized, seeded into 96‐well plates and then calculated with a density of 1 × 10^4^ cells/well. Then, RA FLSs were grown to 80% confluence and were pre‐treated with or without EPZ015666 or transfected with siRNA for 48 hrs and then treated with TNF‐α or IL‐β for 24 hrs. The cell multiplication was detected through a Cell‐Light EdU DNA Cell Proliferation Kit (Roche, Mannheim, Germany). Each test was replicated three times according to the manufacturer's recommendation.

### Confocal laser scanning fluorescence microscopy

RA FLSs or OA FLSs were seeded on sterile cover glass in 35‐mm dishes at a density of 1 × 10^5^ cells/ml. When became approximately 60% confluent, the FLSs were stimulated with IL‐1β or TNF‐α for 24 hrs. Then, they were disposed with paraformaldehyde and infiltrated with PBS containing 0.1% Triton X‐100. The cells were hatched with anti‐PRMT5 antibody all night to measure PRMT5. The cells were then incubated with DAPI, and the coverslips were put on the glass slides with antifade equipment media and then examined by a confocal fluorescence microscopy (Zeiss LSM710, Wetzlar, Germany).

### RNA isolation and quantitative polymerase chain reaction

After the designated treatments with EPZ015666 or transfected with siRNA for 48 hrs and then treated with TNF‐α or IL‐1β for 12 hrs, total RNAs were extracted using TRIzol (Sigma‐Aldrich) and were reverse‐transcribed to cDNA using miScript Reverse Transcription Kit (TaKaRa Biomedical Technology, Kusatsu, Japan ). The mRNA expression of PRMT5, IL‐6 and IL‐8 was analysed by real‐time quantitative polymerase chain reaction (qPCR) which was performed on cDNA using QuantiTect SYBR Green RT‐PCR Kit on StepOnePlusTM Instantaneous analyse PCR System (Applied Biosystems, Foster City, CA, USA). The expression of relative mRNA was more normalized to the GAPDH. The used RT‐PCR primers were listed in Table S1.

### Western blot analysis

For each Western blot experiment, 2 × 10^5^ cells were seeded. When subconfluence (~80%) was reached, RA FLSs were pre‐processed with EPZ015666 for 24 hrs or transfected with siRNA for 48 hrs. Then, RA FLSs were stimulated with or without TNF‐α for 10 min. Cells were lysed with cell lysis buffer (CST) in an ice bath for 15 min., and then, the lysates were centrifuged at 14000 g for 15 min. at 4°C. Supernatants were incubated with 2× laemmli sample buffer (Sigma‐Aldrich) for 5 min. at 100°C. The samples with same amounts were then separated with SDS‐PAGE gel and shifted to NC membranes and immunoblotted with the indicated antibodies: antiphosphor‐AKT (Cell signal transduction), anti‐AKT (Cell signal transduction), anti‐NF‐κB p65 (Cell signalling), antiphosphor‐IKBα (Cell signal transduction), anti‐IKBα (Cell signal transduction) and antiphosphor‐IKK (Cell signal transduction).

### Measurement of inflammatory factors

After the designated treatments with EPZ015666 or transfected with siRNA for 48 hrs and then disposed with TNF‐α for 12 hrs, RA FLSs' (1 × 10^5^ cells/ml, 80% cell confluence) culture supernatants were collected for the measurement of IL‐6 and IL‐8 by enzyme‐linked immunosorbent assay (ELISA), through employing a commercial tool on the basis of the guidance of the manufacturer (R&D Systems, Minneapolis, Minnesota, USA).

### MTT test for FLSs viability

RA FLSs were pre‐processed with EPZ015666 at different concentrations (0.1, 1 and 5 μM) for 48 hrs. The supernatants were removed. The adherent cells were cultured with 3‐(4,5‐dimethylthiazol‐2‐yl)‐2,5‐diphenyltetrazolium salt solution (1 mg/ml in PBS) at 37°C for 30 min. Dark blue formazan was melted in acidified isopropanol, and formazan quantification was performed at a trial wavelength of 570 nm. The wavelength of 620 nm was used as a reference.

### Statistical analysis

The software of SPSS 13.0 (SPSS Inc., Chicago, IL, USA) was utilized. Data were present as means ± standard deviation of the mean (S.E.M.). The differences between each group were detected by *t*‐test or anova. *P* values less than 0.05 were significant.

## Results

### Increased expression of PRMT5 from RA patients

**Figure 1:**
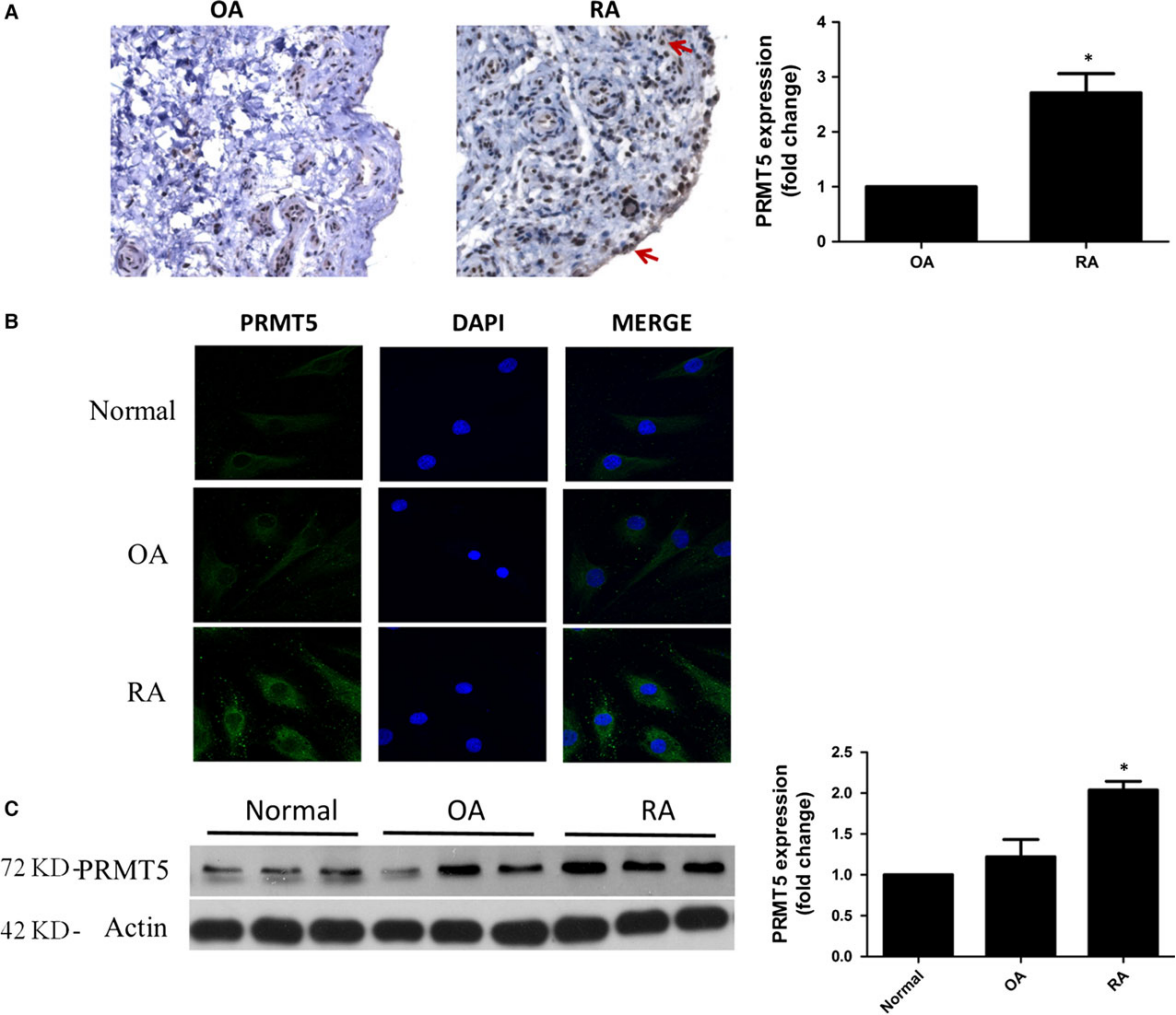
Expression of PRMT5 in patients with RA and OA. (**A**) Representative IHC pictures of PRMT5 expression in STs (left panel). Original magnification 200×. Positive stained cells of anti‐PRMT5 antibody were more prominent in RA than in OA, and most of which were positioned at the sublining layer areas and synovial lining (red arrow). The bar showed the ratio of cells positive for PRMT5 to the total cells (right panel, *n* = 7 patients per group). (**B**) PRMT5 (green) and nuclei (blue) were measured by cellular IF staining. Representative pictures from three independent experiments were shown (*n* = 7 patients per group). Original magnification 630×. (**C**) Protein level of PRMT5 in FLSs was determined by Western blotting (*n* = 7 patients per group). A representative blot of three experiments was shown. *P < 0.05 *versus* OA or normal control.

We first investigated the expression of PRMT5 in STs. Positive stained cells of anti‐PRMT5 antibody were more prominent in STs from RA when compared with OA patients (Fig. 1A). We next examined the expression of PRMT5 in FLSs by IF staining. As a result, RA FLSs exhibited a markedly enhanced staining for PRMT5 and localized in both the cytoplasm and nucleus. Whereas, expression of PRMT5 protein was much less prominent in normal samples and in OA FLSs (Fig. 1B). The constitutive expression of PRMT5 in FLSs was further confirmed by Western blotting. A higher level of PRMT5 expression was detected in RA FLSs than that in OA FLSs or in FLSs from normal controls (Fig. 1C).

### Up‐regulated expression of PRMT5 stimulated with proinflammatory mediators

**Figure 2:**
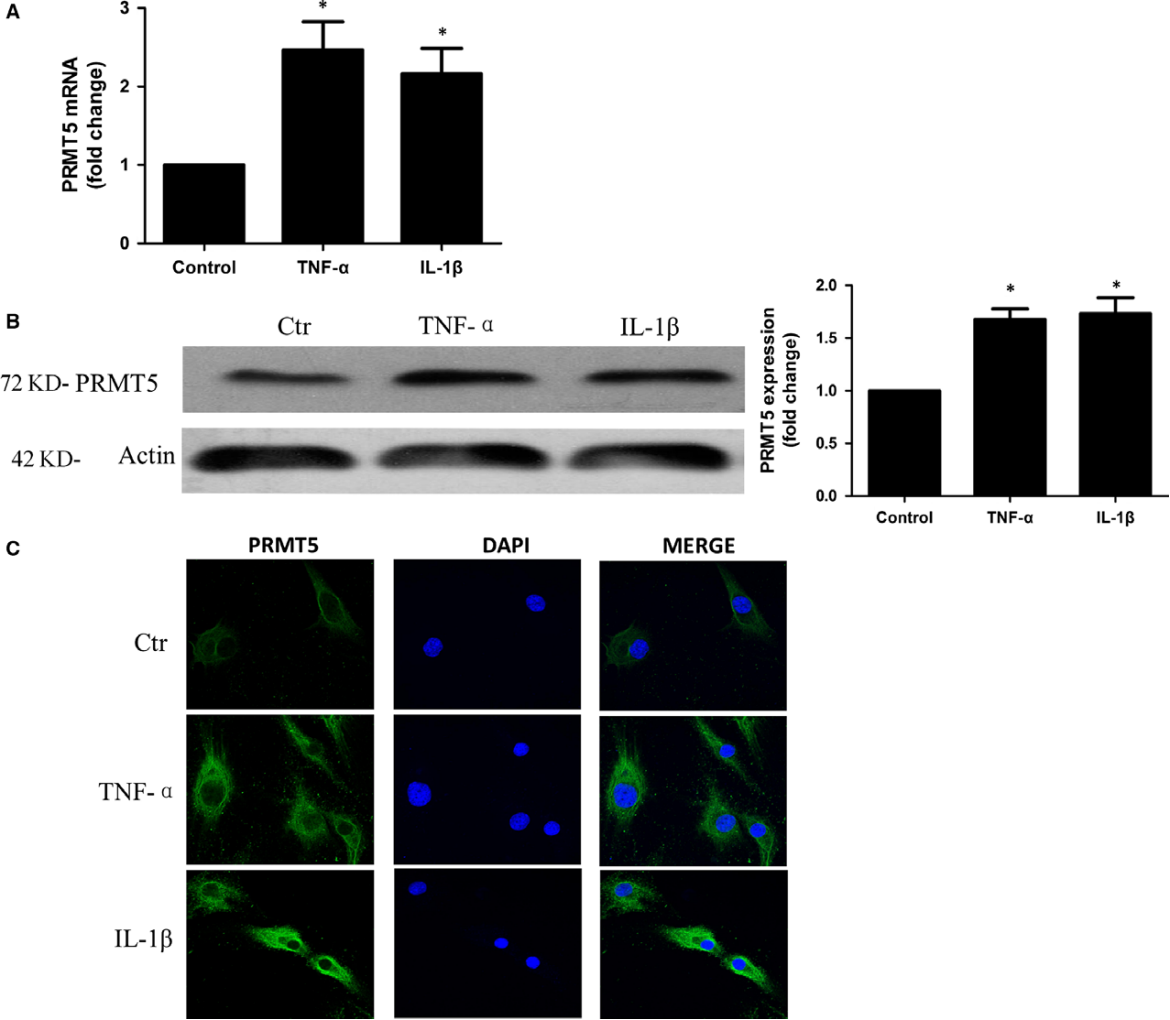
Up‐regulated the expression of PRMT5 by proinflammatory mediators in RA FLSs. Cultured RA FLSs were stimulated with 10 ng/ml TNF‐α or 10 ng/ml IL‐1β. (**A**) Expression of PRMT5 mRNA was analysed by qPCR. Data were normalized with the GAPDH gene as control. (**B**) Expression of PRMT5 in the protein level was measured by Western blotting. Data were presented after normalization by β‐actin (right panel). (**C**) IF staining of anti‐PRMT5 antibody (green) in RA FLSs. The nuclei of cells were counterstained with DAPI (blue). Original magnification 630×. The pictures represented three experiments. Data shown are representative of five independent experiments from three different RA patients. *P < 0.05 *versus* control.

RA FLSs were stimulated with TNF‐α or IL‐1β. As shown in Figures 2A and B, PCR and Western blotting indicated that TNF‐α and IL‐1β significantly enhanced the PRMT5 mRNA expression and the protein level of PRMT5, respectively. The overexpression of PRMT5 in RA FLSs stimulated by proinflammatory mediators was also confirmed by IF staining (Fig. 2C).

### PRMT5 inhibition suppresses production of inflammatory factors and cell proliferation in RA FLSs

**Figure 3:**
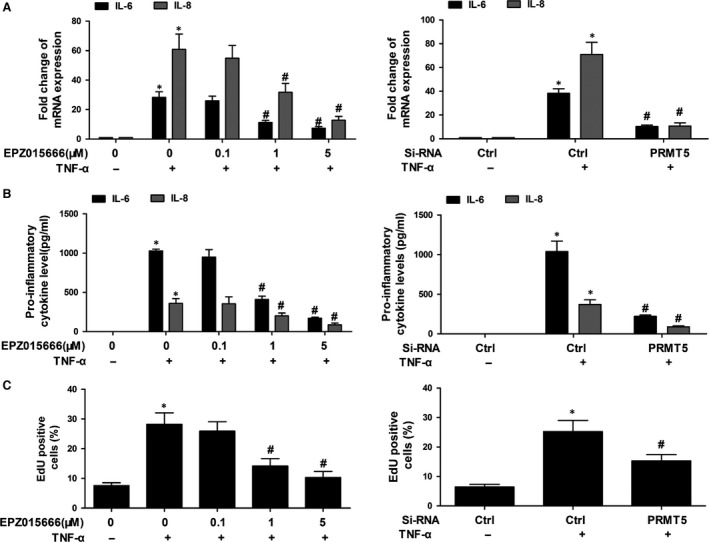
PRMT5 inhibition suppressed the inflammatory factors and cell proliferation in RA FLSs. RA FLSs were pre‐processed with DMSO, or different concentrations of EPZ015666 for 24 hrs, or transfected with PRMT5 siRNA. Then, RA FLSs were stimulated with or without TNF‐α (10 ng/ml) for 12 hrs (for mRNA) or 24 hrs (for ELISA analysis). (**A**) Effect of PRMT5 inhibition on IL‐6 and IL‐8 expression was determined by qPCR. (**B**) The secretion of the IL‐6 and IL‐8 in supernatants was measured by ELISA. (**C**) BrdU incorporation was used to detect cell proliferation. All of the above data represented the mean ± S.E.M. of three experiments from five RA patients. *P < 0.05 *versus* control, ^#^ *P* < 0.05 *versus* TNF‐α.

To investigate the role of PRMT5 on inflammatory response in RA FLSs, a specific PRMT5 inhibitor (EPZ015666) or transfected with PRMT5 siRNA were used. As shown in Figure 3A, TNF‐α‐induced mRNA expression and secretions of IL‐6 and IL‐8 were reduced by treatment with different concentrations of EPZ015666 (0.1~5 μM). Transfection with PRMT5 siRNA also decreased TNF‐α‐induced mRNA and secretions of IL‐6 as well as IL‐8 (Fig. 3B). Moreover, IL‐1β‐induced mRNA expression of IL‐6 and IL‐8 was reduced by treatment with different concentrations of EPZ015666 or transfection with PRMT5 siRNA (Fig. S1A).

Recent studies indicated that inhibition of PRMT5 suppressed proliferation in tumour cell lines. Therefore, we explore whether PRMT5 regulates proliferation of RA FLSs, which was measured by EdU incorporation. We showed that pre‐treatment with EPZ015666 and PRMT5 siRNA reduced TNF‐α‐induced proliferation of RA FLSs (Fig. 3C). Furthermore, pre‐treatment with EPZ015666 and PRMT5 siRNA also reduced IL‐1β‐induced proliferation of RA FLSs (Fig. S1B).

MTT test was used to evaluate the toxic effect of EPZ015666 on RA FLS viability. Up to 5 μM, EPZ015666 did not reduce the viability of RA FLSs, which indicated that the inhibitory effects of EPZ015666 were not as a result of drug cytotoxicity (data not shown).

### PRMT5 inhibition suppresses the migration and invasion of RA FLSs

**Figure 4:**
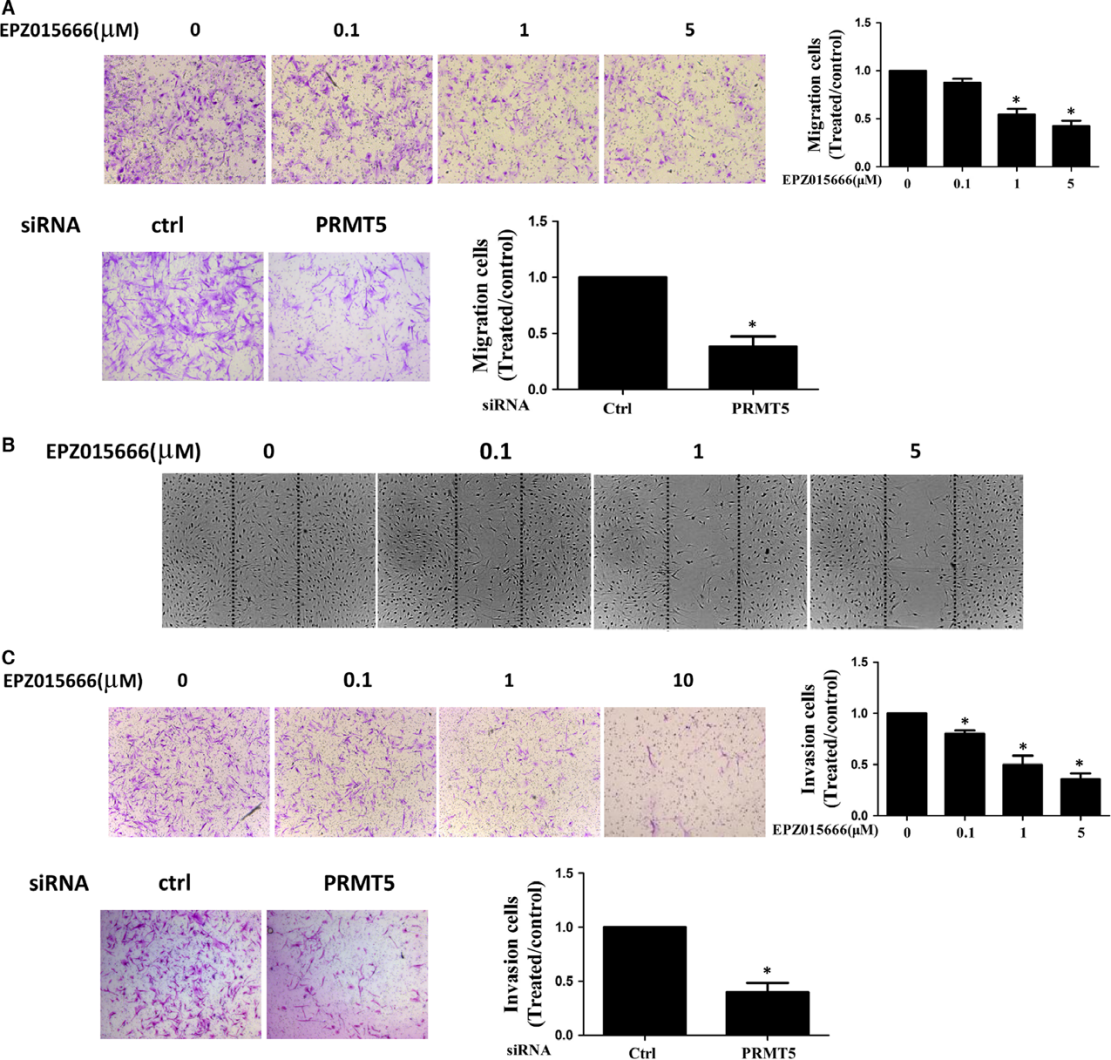
Inhibition of PRMT5 decreased migration and invasion of RA FLSs. RA FLSs pre‐treated with DMSO as control, various concentrations of EPZ015666 or transfected with PRMT5 siRNA or control siRNA were serum starved overnight at 24 hrs. (**A**) Migration was performed in a Boyden chamber (original magnification 100×). Photo images were representative of migration of RA FLSs treated with EPZ015666 (upper panel) or transfected with PRMT5 siRNA or control siRNA (ctrl) (lower panel). (**B**) Effect of different concentrations of EPZ015666 on the wounding migration of RA FLSs (original magnification ×100). (**C**) Invasion was performed in a Matrigel basement membrane matrix chamber. Photo images were representative of invasion of RA FLSs treated with EPZ015666 (upper panel) or transfected with PRMT5 siRNA or control siRNA (ctrl) (lower panel). All data were representative of experiments from five patients with RA. *P < 0.05 *versus* control.

Then, we explored the role of PRMT5 in the invasion and migration of RA FLSs using a specific PRMT5 inhibitor (EPZ015666) or PRMT5 siRNA transfection. Treatment with EPZ015666 and PRMT5 siRNA markedly inhibited FBS‐induced migration of RA FLSs (Fig. 4A). Wound closing was significantly slowed in RA FLSs pre‐treated with EPZ015666 and transferred with PRMT5 siRNA (Fig. 4B). We also observed that treatment with EPZ015666 and PRMT5 siRNA suppressed invasion of RA FLSs (Fig. 4C). The above results revealed that PRMT5 played a crucial role in regulating RA FLSs migration and invasion.

### PRMT5 inhibition regulates NF‐κB activation through IκB kinase in RA FLSs

**Figure 5:**
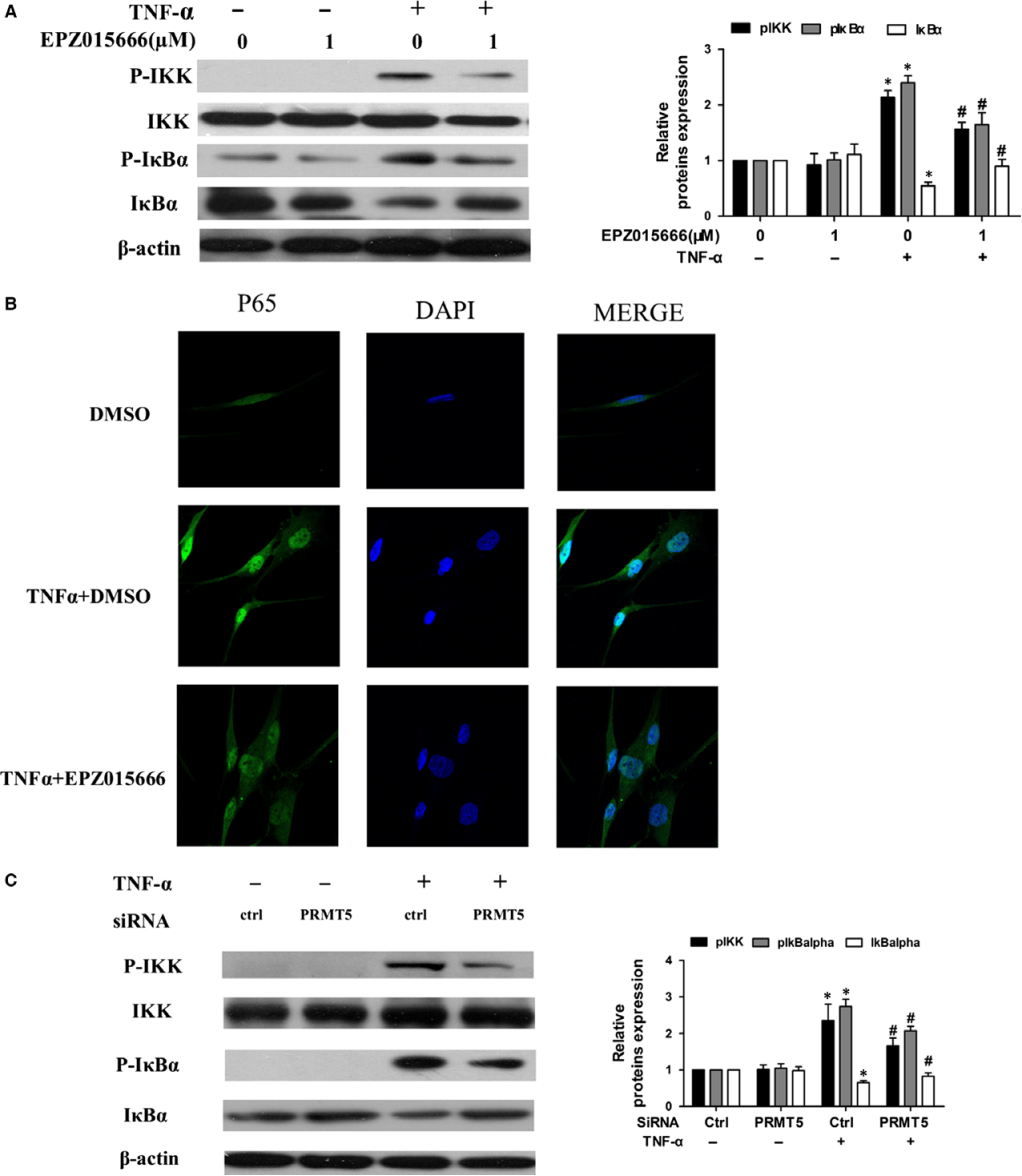
Effect of PRMT5 inhibition on NF‐κB in activated RA FLSs. (**A**) RA FLSs were pre‐processed with DMSO as control or with EPZ015666 for 24 hrs. Then, RA FLSs were stimulated by TNF‐α (10 ng/ml) for 15 min. The protein levels of p‐IKK, total‐IKK, p‐IκBα and total IκBα were analysed by Western blot. Shown was the representative blot of three experiments. (**B**) Effect of EPZ015666 on nuclear translocation of p65. Middle panel showed a representative photo of IF staining of p65 localization (green). Nuclei were stained with DAPI. (**C**) Western blot analysis for p‐IKK, total‐IKK, p‐IκBα and total IκBα following TNF‐α stimulation was shown in cells transfected with PRMT5 siRNA. A representative blot of three independent experiments was shown. All values represented mean ± S.E.M. from five different RA patients. *P < 0.05 *versus* control. ^#^ *P* < 0.05 *versus* TNF‐α stimulation.

Under proinflammatory stimulation, activation of NF‐κB pathway required the activation of IκB kinase (IKK). Activated IKK promoted the phosphorylation and degradation of the inhibitor of NF‐κB (IκB), and phosphorylation of p65. We further explore the association between PRMT5 and NF‐κB. As shown in Figure 5A, a specific inhibitor of PRMT5 decreased phosphorylated IKK (pIKK), as well as phosphorylation and degradation of IκBα in TNF‐α‐stimulated RA FLSs. We then observed EPZ015666 decreased p65 nuclear accumulation (Fig. 5B). Furthermore, we observed that transfection with PRMT5 siRNA suppressed the TNF‐α‐induced pIKK and phosphorylation of IκBα (Fig. 5C). These data suggested that inhibition of PRMT5 suppressed NF‐κB activation through IKK signalling.

### PRMT5 inhibition regulates TNF‐α‐induced activation of AKT in RA FLSs

**Figure 6:**
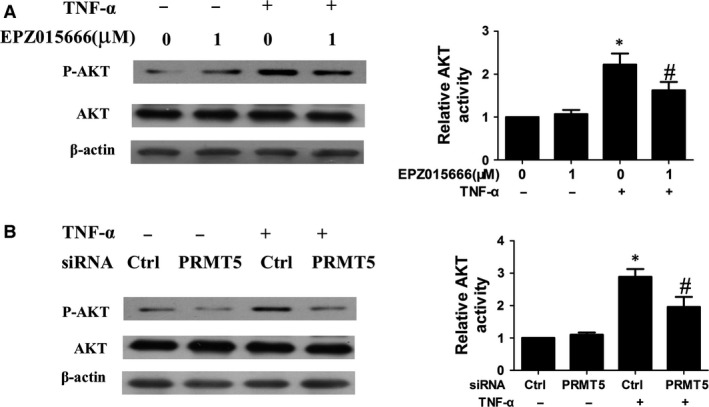
Effect of PRMT5 inhibition on TNF‐α‐induced activation of AKT in RA FLSs. (**A** and **B**) Western blot analysis of phosphorylated AKT in RA FLSs treated with EPZ015666 for 24 hrs or transfected with PRMT5 siRNA for 48 hrs followed by TNF‐α (10 ng/ml) for 10 min. Densitometry was performed, and fold change of protein expression was shown (right panel). All values represented as mean ± S.E.M. from 5 RA patients. *P < 0.05 *versus* control or control siRNA, ^#^ *P* < 0.05 *versus* TNF‐α.

Because AKT was an important factor in regulating inflammation and migration of RA FLSs, we further investigated the relationship between PRMT5 and AKT. It was observed that EPZ015666 treatment inhibited phosphorylation of AKT in TNF‐α‐treated RA FLSs (Fig. 6A). We also observed the similar results from RA FLSs transfected with PRMT5 siRNA (Fig. 6B). These data suggested that AKT might be involved in PRMT5‐mediated inflammation and migration of RA FLSs.

## Discussion

Our results demonstrated that RA FLSs constitutively express PRMT5, which was up‐regulated by stimulation with proinflammatory cytokines. Moreover, inhibition of PRMT5 suppressed the production of IL‐6 and IL‐8 and prevented cell proliferation, migration and invasion by attenuating the activation of NF‐κB and AKT in RA FLSs. Taken together, the data suggested a crucial role of PRMT5 in regulating inflammation and the maintenance of an activated FLSs phenotype in patients with RA. Inhibition of PRMT5 might be a hopeful target for the therapy of RA.

Pro‐inflammatory cytokines initiated synovial inflammation in RA. However, the mechanisms that modulated the production of pro‐inflammatory cytokines in RA remained to be identified. Here, we demonstrated that PRMT5 inhibition decreased TNF‐α‐induced expression of IL‐6 and IL‐8 in RA FLSs. Recent studies suggested that PRMT5‐mediated post‐translational modifications might be associated with inflammatory. PRMT5 methylated a key transcriptional factor HOXA9 of endothelial cell inflammatory responses, which was critical in up‐regulation of endothelial–leucocyte adhesion molecules 22, 23. PRMT5‐medicated arginine methyltransferase was indicated as an important step for CXCL10 transcription in TNF‐α‐activated human endothelial cells inflammation. Our data indicated one of the mechanisms of PRMT5 in the maintenance of the activated phenotype of RA FLSs via regulation the inflammatory response.

The invasive behaviours of RA FLSs to the cartilage and bone were essential steps in the aggravation of RA 6, 24, 25. PRMT5 was the major enzyme responsible for arginine dimethylation, which was associated with tumour cell growth in different types of cancer 15, 16, 17, 18, 19. In this study, we found that inhibition of PRMT5 strongly reduced cell proliferation, migration and invasion. Consistent with our findings, Zhang *et al*. and Guo *et al*. 26, 27 reported that treatment with a PRMT5 inhibitor decreased migratory activity in several tumour cell lines. As abnormal migration and invasion of RA FLSs were critical in destruction of joints, our results provided new evidence on the association between PRMT5 and the aggressive nature of RA FLSs.

We observed that PRMT5 inhibition suppressed TNF‐α‐induced phosphorylation of IKKβ and IκBα, as well as translocation of nuclear NF‐κB. These data indicated that PRMT5 regulated the NF‐κB pathway by interfering in early cytoplasmic IKK signalling. Similar findings were also indicated in other studies. According to Wei *et al*. 28 *,* PRMT5 methylated Arg‐30 (R30) residues of the p65 subunit, and consequently regulated NF‐κB‐dependent gene expression, such as IL‐1α and TNF receptor‐associated factor 1. Additionally, it has also been reported that R35 as well as R30 at p65, which was methylated by PRMT5, participated in transcription of TNF‐α‐induced pro‐inflammatory genes in endothelial cells 22.

Simulated by TNF‐α, phosphoinositide AKT was crucial in regulating cell proliferation and inflammation in RA 29. We found that inhibition of PRMT5 leaded to decreased expression of p‐AKT, without affecting total AKT. Our results were also supported by studies from tumour cells. Wei *et al*. 30 demonstrated that inhibition of PI3K/AKT activity could block PRMT5‐induced cell proliferation in lung cancer cell lines, including A549 and H1299. Our finding identified a new pathway linking PRMT5 to AKT activation.

In conclusion, we showed that PRMT5 played an important role in inflammatory response, cell proliferation, migration and invasion of RA FLSs through NF‐κB and AKT pathway. Thus, inhibition of PRMT5 might be a potential therapeutic agent to attenuate pathological progression in rheumatoid arthritis.

## Conflict of interest

The authors confirm that there are no conflict of interests.

## Supporting information


**Figure S1** PRMT5 inhibition suppressed the inflammatory factors and cell proliferation in IL‐1β RA FLSs.

**Table S1** The sequences of RT‐PCR primers.
