# Supplementary figures and images for: Role of protein arginine methyltransferase 5 in inflammation and migration of fibroblast‐like synoviocytes in rheumatoid arthritis
Source: J Cell Mol Med. 2016 Nov 17;21(4):781–90. doi: 10.1111/jcmm.13020 (PMC5345686; doi:10.1111/jcmm.13020)

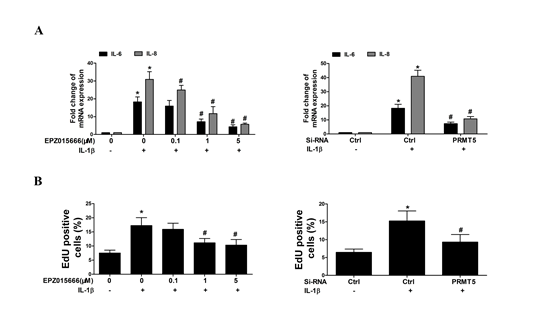

Supplement: Supplementary file 1 — Figure S1 PRMT5 inhibition suppressed the inflammatory factors and cell proliferation in IL‐1β RA FLSs. [file JCMM-21-781-s001.tif]
