# Supplementary material for: Role of protein arginine methyltransferase 5 in inflammation and migration of fibroblast‐like synoviocytes in rheumatoid arthritis
Source: J Cell Mol Med. 2016 Nov 17;21(4):781–90. doi: 10.1111/jcmm.13020 (PMC5345686; doi:10.1111/jcmm.13020)
Supplement: Supplementary file 2 — Table S1 The sequences of RT‐PCR primers. [file JCMM-21-781-s002.doc]

Table S1 The sequences of RT-PCR primers

|  | Sequences | |
| --- | --- | --- |
| PRMT5 | Forward | CTGTCTTCCATCCGCGTTTCA |
|  | Reverse | GCAGTAGGTCTGATCGTGTCTG |
| IL6 | Forward | ACTCACCTCTTCAGAACGAATTG |
|  | Reverse | CCATCTTTGGAAGGTTCAGGTTG |
| IL8 | Forward | ACTGAGAGTGATTGAGAGTGGAC |
|  | Reverse | AACCCTCTGCACCCAGTTTTC |
